# Supplementary figures and images for: Genomic selection models double the accuracy of predicted breeding values for bacterial cold water disease resistance compared to a traditional pedigree-based model in rainbow trout aquaculture
Source: Genet Sel Evol. 2017 Feb 1;49:17. doi: 10.1186/s12711-017-0293-6 (PMC5289005; doi:10.1186/s12711-017-0293-6)

## Slide 1
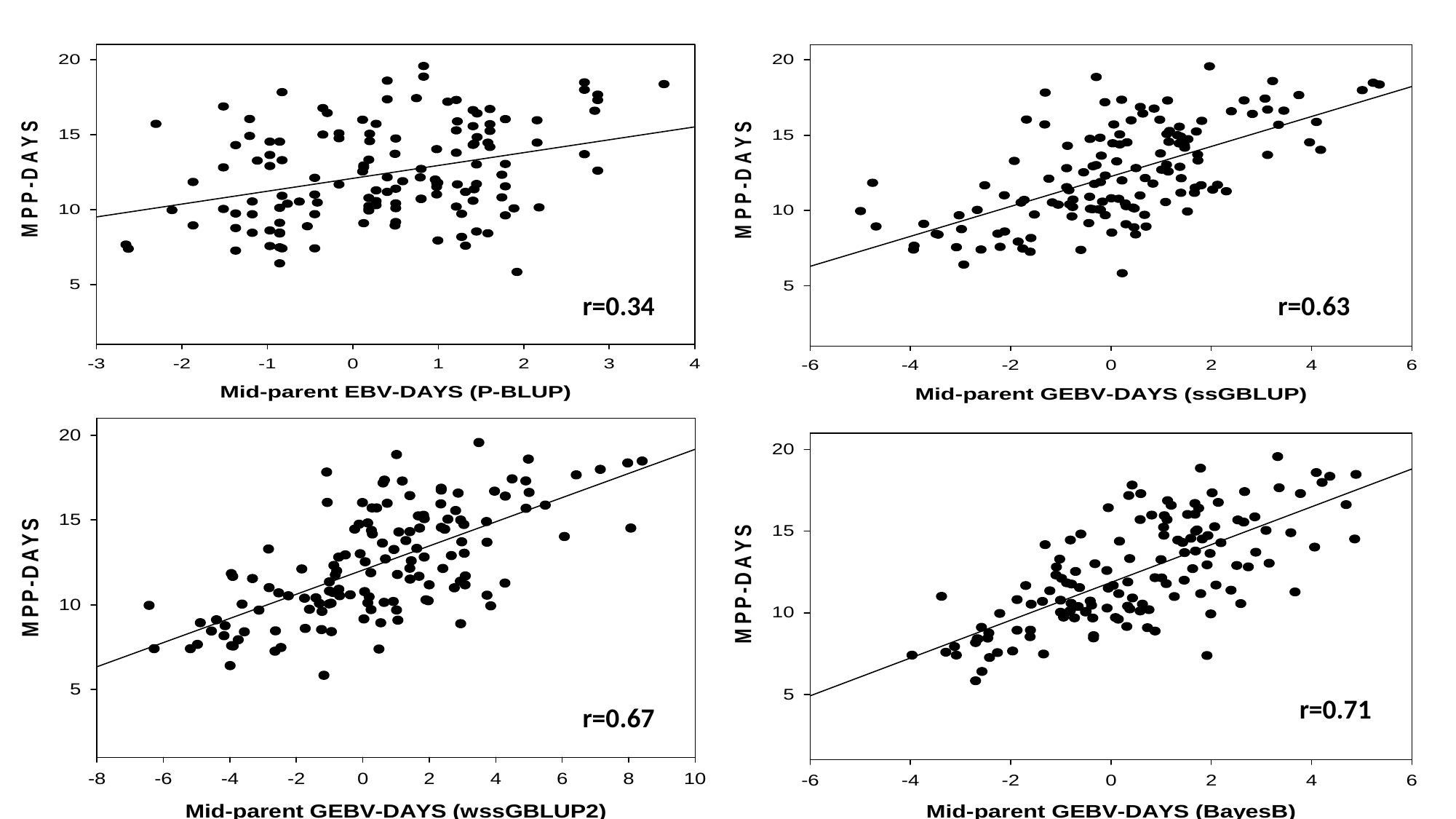

r=0.34
r=0.63
r=0.71
r=0.67

Supplement: Supplementary file 2 — Additional file 2: Figure S1. Correlation between mean progeny phenotype (MPP) and mid-parent EBV or GEBV for BCWD survival DAYS from progeny testing families (PTF). This figure represents the correlation between mean progeny phenotype (MPP) and mid-parent EBV or GEBV for BCWD survival DAYS from 138 progeny testing families (PTF) of rainbow trout. [file 12711_2017_293_MOESM2_ESM.pptx]

## Slide 1
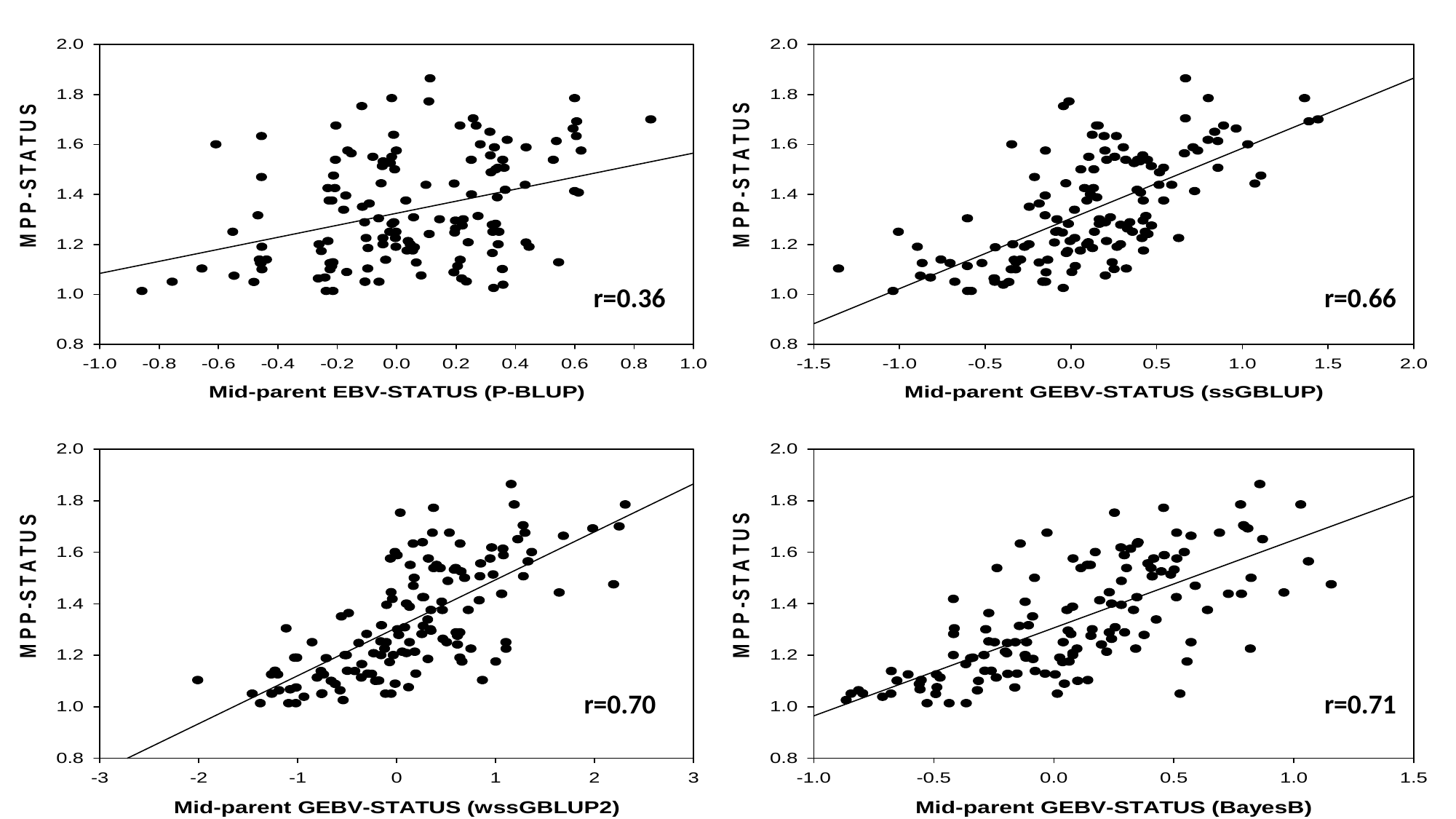

r=0.66
r=0.36
r=0.70
r=0.71

Supplement: Supplementary file 3 — Additional file 3: Figure S2. Correlation between mean progeny phenotype (MPP) and mid-parent EBV or GEBV for BCWD survival STATUS from progeny testing families (PTF). This figure represents the correlation between mean progeny phenotype (MPP) and mid-parent EBV or GEBV for BCWD survival STATUS from 138 progeny testing families (PTF) of rainbow trout. [file 12711_2017_293_MOESM3_ESM.pptx]
